# *In Vitro* Effects of Bisphenol A** β**-D-Glucuronide (BPA-G) on Adipogenesis in Human and Murine Preadipocytes

**DOI:** 10.1289/ehp.1409143

**Published:** 2015-05-27

**Authors:** Jonathan G. Boucher, Adèle Boudreau, Shaimaa Ahmed, Ella Atlas

**Affiliations:** Environmental Health Science and Research Bureau, Health Canada, Ottawa, Ontario, Canada

## Abstract

**Background:**

Exposure to common environmental substances, such as bisphenol A (BPA), has been associated with a number of negative health outcomes. *In vivo*, BPA is rapidly converted to its predominant metabolite, BPA-glucuronide (BPA-G), which has long been believed to be biologically inactive because it lacks estrogenic activity. However, the effects of BPA-G on cellular metabolism have not been characterized. In the present study we examined the effect of BPA-G on adipogenesis.

**Methods:**

The effect of BPA-G on the differentiation of human and 3T3L1 murine preadipocytes was evaluated *in vitro* by quantifying lipid accumulation and the expression of adipogenic markers.

**Results:**

Treatment of 3T3L1 preadipocytes with 10 μM BPA-G induced a significant increase in lipid accumulation, mRNA expression of the adipogenic markers sterol regulatory element binding factor 1 (*SREBF1*) and lipoprotein lipase (*LPL*), and protein levels of LPL, aP2, and adipsin. Treatment of primary human preadipocytes with BPA-G also induced adipogenesis as determined by aP2 levels. Co-treatment of cells with the estrogen receptor (ER) antagonist fulvestrant (ICI) significantly inhibited the BPA-G–induced increase in LPL and aP2 levels, whereas treatment with ICI alone had no effect. Moreover, BPA-G did not display any significant estrogenic activity.

**Conclusions:**

To our knowledge, this study is the first to report that BPA-G induces adipocyte differentiation and is not simply an inactive metabolite. The fact that BPA-G induced adipogenesis and was inhibited by an ER antagonist yet showed no estrogenic activity suggests that it has no classical ER transcriptional activation function and acts through a pathway that remains to be determined.

**Citation:**

Boucher JG, Boudreau A, Ahmed S, Atlas E. 2015. *In vitro* effects of bisphenol A β-D-glucuronide (BPA-G) on adipogenesis in human and murine preadipocytes. Environ Health Perspect 123:1287–1293; http://dx.doi.org/10.1289/ehp.1409143

## Introduction

Environmental chemicals with widespread human exposure, such as bisphenol A (BPA), may play an important role in deregulating normal metabolism (vom Saal et al. 2012). BPA is used in the manufacture of polycarbonate plastic (Fleisch et al. 2010; Rubin 2011) and has been detected in food, water, and dust (Vandenberg et al. 2007). Human exposure to BPA is demonstrated by its ubiquitous detection in blood, urine, and adipose tissue (Dekant and Völkel 2008; Vandenberg et al. 2010), and it has been linked to modulation of adipocyte differentiation and obesity (Bhandari et al. 2013; Harley et al. 2013; Masuno et al. 2005; Somm et al. 2009; Wang et al. 2013).

*In vivo*, BPA is rapidly conjugated to glucuronide, giving rise to BPA-glucuronide (BPA-G) after passing through the liver (Knaak and Sullivan 1966; Pottenger et al. 2000). The metabolism of BPA in rat, monkey, and human hepatocytes displays some species specificity; however, BPA-G has been shown to be BPA’s major metabolite (Kurebayashi et al. 2010). BPA-G is excreted primarily in the urine in humans and the bile in rodents (Inoue et al. 2001, 2003; Völkel et al. 2002). BPA-G has been detected in human serum and urine at higher concentrations than free BPA itself (Harthé et al. 2012; Liao and Kannan 2012; Smith and Saggerson 1979). In addition, BPA-G but not free BPA was detected in blood of humans exposed to low doses of BPA (Völkel et al. 2002). BPA-G has also been investigated in a number of animal models. In rats orally administered BPA (100 mg/kg) over 6 days, the major form of BPA in the urine, milk, and plasma was BPA-G (Snyder et al. 2000). Moreover, pregnant sheep exposed to BPA showed a 1,300-fold higher BPA-G accumulation in amniotic fluid and cord blood compared with BPA (Viguié et al. 2013).

It has been the long-held belief that BPA-G, the predominant metabolite of BPA exposure, has no bioactivity and is quickly eliminated *in vivo*. This is mainly due to the fact that BPA is well-known for its estrogenic activity, whereas BPA-G has been shown to lack the ability to activate the estrogen receptor (ER) (Matthews et al. 2001). In HepG2 human hepatoma cells, ERα and ERβ activity was not significantly affected after exposure to BPA-G (Snyder et al. 2000). However, free BPA has been shown to possess bioactivity beyond estrogenicity, and therefore it would be reasonable to assume that BPA-G could potentially influence alternate pathways much like BPA does. We and others have previously shown that BPA is a significant inducer of adipogenesis in both rodent and human cell models, independent of its estrogenic activity (Boucher et al. 2014; Sargis et al. 2010). BPA has been linked to activation of the glucocorticoid receptor (GR) as well as the thyroid receptor (TR), androgen-receptor (AR), and estrogen-receptor-related (ERR) γ (Lee et al. 2003; Matsushima et al. 2007; Moriyama et al. 2002; Sargis et al. 2010). The effect of BPA-G on anything other than estrogenic activity and on other receptors has not been determined.

In the present study, the effect of BPA-G on the differentiation of the murine 3T3L1 preadipocyte cell line and primary human preadipocytes was examined. We observed that BPA-G was not an inactive metabolite but was able to induce lipid accumulation in both human and murine preadipocytes and to increase expression of several key adipogenic markers at the mRNA and protein levels. Moreover, BPA-G–induced differentiation was inhibited in the presence of the specific ER antagonist fulvestrant (ICI-182780; ICI), despite the fact that BPA-G has no estrogenic activity in these cells, suggesting that a potential mechanism of BPA-G action may be through a nonclassical ER action or as yet unknown pathway.

## Materials and Methods

*Adipocyte differentiation.* 3T3L1 mouse embryo fibroblasts (ATCC) were cultured according to the supplier’s instructions and were not used past passage 10. Cells were cultured in Dulbecco’s Modified Eagles Medium (DMEM)/low glucose media containing 10% bovine calf serum (HyClone Laboratories). Two days after reaching confluence, cells were cultured in differentiation medium consisting of DMEM, 10% fetal bovine calf serum (FBS), 500 μM 3-isobutyl-1-methylxanthine (IBMX) (Sigma-Aldrich), 100 nM insulin (Roche Diagnostics), and varying concentrations of BPA-G (Toronto Research Chemicals Inc.) or 250 nM dexamethasone (DEX) (Sigma-Aldrich). Media containing insulin with or without BPA-G were replenished every 2 days. For the ER and GR antagonist studies, 0.01–10 μM ICI or 1 μM of the GR antagonist RU486 or 1 nM estradiol (E2) (all from Sigma-Aldrich) were also added to the cells with or without BPA-G. Primary human preadipocytes (Zenbio Inc.) from donors with body mass indexes < 24.99 and who gave informed consent were maintained in Preadipocyte Medium (ZenBio). For differentiation, confluent preadipocytes were treated with media containing 33 μM biotin, 17 μM pantothenate (Sigma-Aldrich), and 100 nM insulin for 14 days. In addition, 500 μM IBMX was also included in the differentiation media from day 0 to day 4. From day 2 until day 14, 5 μM troglitazone (Sigma-Aldrich) and the indicated concentrations of BPA-G or 1 μM DEX (as a positive control) were also included in the differentiation media and replenished every 2 days. Ethics approval for the use of primary human adipocytes was obtained from the Health Canada Research Ethics Board.

*Lipid staining and quantification*. Clear-bottomed black 96-well plates were coated with rat collagen I (Invitrogen) at 5 μg/cm^2^ in 0.02 M acetic acid for 1 hr at room temperature. 3T3L1 cells were seeded and differentiated as described above for 8 days with the indicated treatments. Cells were then fixed with 10% formalin and stained with Nile red and DAPI (both 1 μg/mL) in 0.2% Triton-phosphate-buffered saline (PBS) for 15 min as previously described (Greenspan et al. 1985). Nile red (staining for lipid droplets) was viewed at 530 nm and DAPI (staining for cell nuclei) at 405 nm using fluorescence imaging on an Olympus IX71 microscope. Cells were imaged at 100× magnification. Nile red fluorescence was quantified at 485/528 nm (excitation/emission) and normalized to DAPI staining measured at 360/460 nm using a Synergy 2 Microplate Reader (BioTek Instruments Inc.).

*Real-time polymerase chain reaction (PCR)*. We used the the RNeasy Kit (Qiagen) to extract total RNA from differentiating cells treated as described above. Genomic DNA was eliminated using the RNase-Free DNase Kit (Qiagen). RNA (250–500 ng) was reverse transcribed into cDNA using iScript Advanced cDNA Synthesis Kit (BioRad). For each real-time PCR reaction, cDNA was amplified in a CFX96-PCR Detection System using the iQ SYBR SsoFast EvaGreen Supermix (BioRad). The primer pairs for each gene target were *C/EBP* α: (CCAAT/enhancer-binding protein α): forward-TAAC​TCCC​CCAT​GGAG​TCGG, reverse-TATA​GACG​TCTC​GTGC​TCGC; *LPL* (lipoprotein lipase): forward-GATC​CGAG​TGAA​AGCC​GGAG, reverse-TTGT​TTGT​CCAG​TGTC​AGCC​A; *SREBF1* (sterol regulatory element binding factor 1): forward-CTTT​TCCT​TAAG​GTGG​GCCT, reverse-AGCT​GGAG​CATG​TCT​TCGA​T; *PPAR*γ 1/2 (peroxisome proliferator-activated receptor γ 1/2): forward-GCCT​GCGG​AAGC​CCTT​TGGT, reverse-GCAG​TTCC​AGGG​CCTG​CAGC; *aP2*: forward-GGAA​GCTT​GTCT​CCAG​TGAA, reverse-GCGG​TGAT​TTCA​TCGA​ATTC; *Adipsin*: forward-CCTG​AACC​CTAC​AAGC​GATG, reverse-CAAC​GAGG​CATT​CTGG​GATA​G; *Perilipin*: forward-TTGG​GGAT​GGCC​AAAG​AGAC, reverse-CTCA​CAAGG​CTTG​GTTT​GGC; and β*-actin*: forward-GACT​TCGA​GCAA​GAGA​TGGC, reverse-CCAG​ACAG​CACTG​TGTTG​GC. Standard curves were generated from the pooled cDNA obtained from cells treated with DEX from various time points. Primer efficiencies were > 90%, and specificity was confirmed by sequence blast and melting curve analysis. Reactions were normalized to β-actin expression, which was not affected by BPA-G treatment.

*Western blot analysis*. Cells were lysed in RIPA buffer (20 mM Tris, pH 7.5, 150 mM NaCl, 1 mM EDTA, 1% sodium deoxycholate, 2% NP-40, 0.4% SDS, 10% glycine) containing protease inhibitors (Roche Diagnostics). Primary antibodies for aP2 and LPL (both from R&D Systems), adipsin (Santa Cruz Biotechnology), and β-actin (13E5; Cell Signaling Technology), as well as appropriate horseradish peroxidase–labeled secondary antibodies were used. Blots were developed using Clarity Western ECL Substrate (BioRad). Western blots were visualized usingChemiDoc Imager and quantified using Image Lab software (BioRad). Protein levels were normalized using β-actin.

*ER and GR transcription assays*. 3T3L1 cells were plated in triplicate in 12-well cell culture plates in 10% charcoal stripped serum (Wisent) and phenol red–free DMEM (Wisent) media. After 24 hr, the cells were transfected with 3X ERE (estrogen-responsive element)–TATA-luciferase reporter plasmid (ERE-Luc) and ERα and ERβ expression plasmids (pVP16-ERα and pVP16-ERβ, respectively) (all from Addgene) using Fugene HD (Roche Diagnostics) according to the manufacturer’s instructions. The cells were transfected with 125 ng of the reporter plasmid, 25 ng ER-expression plasmid, and 10 ng pRL-CMV (Promega) used as an internal control. For the GR transcription assays, experiments were performed in Cos-7 cells, which were transfected as described above with either 125 ng 3X-GRE-luc (GR-responsive element) or 125 ng aP2-luc (plasmid containing the aP2 promoter region upstream of luciferase (Atlas et al. 2014), 25 ng pTL2-GR (a GR expression plasmid), and 10 ng pRL-CMV as an internal control. Twenty-four hours after transfection, cells were treated with ethanol (vehicle control), 10 μM BPA-G, 250 nM DEX, 1 μM RU486 (GR antagonist), or co-treatment conditions for 24 hr. Cells were then lysed in Passive Lysis Buffer (Promega), and luciferase activity was quantified with the Dual Luciferase Assay kit (Promega) using a Glomax96 Luminometer (Promega). Luciferase activity was normalized to the internal control.

*Statistical analyses.* Data were analyzed by Student’s *t*-test or analysis of variance (ANOVA) with Holm-Sidak post-test analysis using SigmaPlot 11.0 (Systat Software Inc.).

## Results

**Figure 1:**
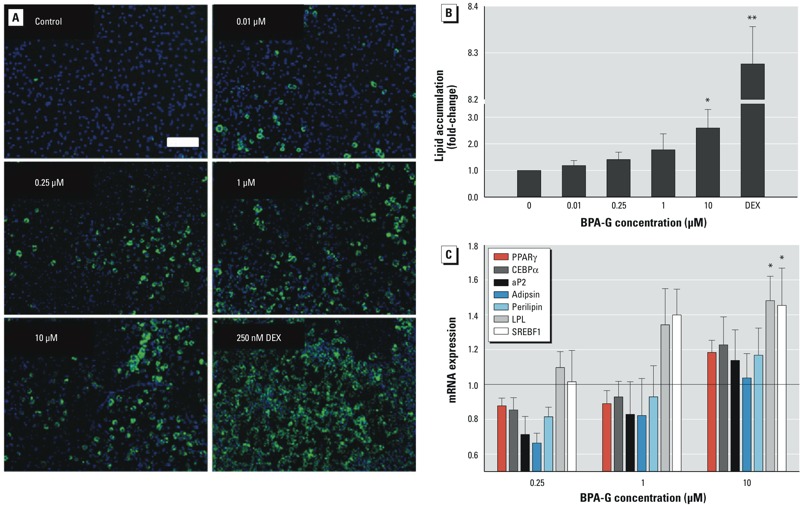
Effects of bisphenol A-glucuronide (BPA-G) on lipid accumulation and adipogenic gene expression in 3T3L1 preadipocytes. Murine 3T3L1 cells were treated with ethanol (control), BPA‑G (0.01–10 μM), or 250 nM dexamethasone (DEX; positive control). (*A,B*) Lipid accumulation on day 8 of differentiation was visualized using fluorescent Nile red staining (*A*; bar = 200 μm) and quantified using a microplate reader (*B*); each culture was performed in triplicate. (*C*) 3T3L1 preadipocytes were treated with increasing concentrations of BPA‑G; total RNA was isolated on day 6 posttreatment and used for quantitative real-time PCR analysis of the adipogenic markers *PPAR*γ, *CEBP*α, *aP2, Adipsin, Perilipin, LPL, *and *SREBF1*. Values were normalized to β-actin gene expression and are expressed as mean fold change relative to control ± SEM for four experiments. *p < 0.05, and **p < 0.001 relative to control by one-way ANOVA with Holm-Sidak post-test analysis.

*Effects of BPA-G on lipid accumulation and expression of adipogenic markers in 3T3L1 preadipocytes*. We evaluated the effect of BPA-G on adipocyte differentiation by assessing lipid accumulation using Nile red staining and quantification. 3T3L1 cells treated with 0.01–10 μM BPA-G for 8 days showed increased Nile red lipid staining, indicating more differentiation compared with control cells treated with vehicle alone (ethanol) (Figure 1A). In cells treated with DEX as a positive control, roughly 90–100% of the cells were positive for lipid staining, indicating a high degree of adipocyte differentiation. Quantification of Nile red staining showed significant lipid accumulation at 10 μM BPA-G (Figure 1B), inducing a 3-fold increase in fluorescence levels relative to control. Cells treated with DEX exhibited an almost 8.3-fold increase in lipid accumulation. No cell death was observed under any of the treatment conditions. To further evaluate BPA-G–induced effects on differentiation, we used quantitative real-time PCR to evaluate mRNA expression of key adipogenic factors on day 6 posttreatment in response to increasing doses of BPA-G (0.25–10 μM). Treatment of cells with 10 μM BPA-G resulted in a statistically significant increase in the mRNA levels of the adipogenic markers *SREBF1* and *LPL* by 1.5-fold the level of control (Figure 1C). We also measured the mRNA expression of several adipogenic markers throughout the course of differentiation at days 2, 4, 6, and 8 for all doses of BPA-G, as well as for 250 nM DEX (positive control), and observed modest increases in *LPL* and *SREBF1* mRNA levels by day 6 after BPA-G treatment (see Supplemental Material, Figure S1). The data show that BPA-G can induce adipocyte differentiation and lipid accumulation in a time-dependent manner.

**Figure 2:**
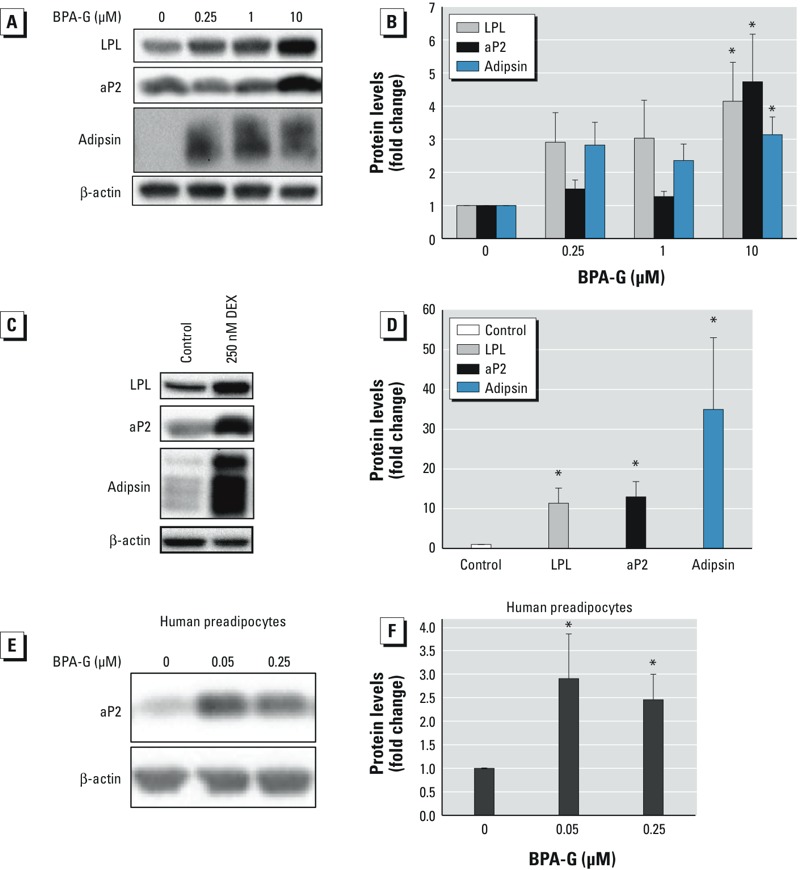
Effect of BPA‑G treatment on protein levels of adipogenic markers in murine 3T3L1 preadipocytes (*A–D*) and primary human preadipocytes (*E,F*). (*A,B*) Murine 3T3L1 preadipocytes were differentiated with BPA‑G for 8 days, and protein levels of the adipogenic markers LPL, aP2, and adipsin were assessed by Western blot (*A*) and densitometry (*B*). (*C,D*) 3T3L1 cells were treated for 8 days with ethanol (control) or 250 nM DEX as a positive control, and protein levels of the adipogenic markers were assessed by Western blot (*C*) and densitometry (*D*). (*A,C*) β-Actin was used as the protein loading control, and images are representative of at least three separate experiments. (*B,D*) Densitometry values are expressed as mean ± SEM of at least six experiments. (*E,F*) Primary human preadipocytes were treated with ethanol (control) or BPA‑G, and the protein levels of the adipogenic marker aP2 were assessed by Western blot (*E*) and densitometry (*F*) at day 14 of differentiation. (*E,F*) β-Actin was used as the gel loading control, and values are expressed as mean ± SEM of at least four separate experiments. *p < 0.05 relative to control, by one-way ANOVA with Holm-Sidak post-test analysis.

*Effects of BPA-G on protein expression of adipogenic markers*. The effects of BPA-G treatment on the protein levels of the adipogenic markers LPL, aP2, and adipsin were evaluated. The data show that levels of these three proteins were significantly increased on day 8 only with 10 μM BPA-G and DEX relative to the control (Figure 2A–D). The effect of BPA-G on the differentiation of primary human preadipocytes was also evaluated. The data showed that treatment of cells with 0.05 and 0.25 μM significantly stimulated adipocyte differentiation, as determined by aP2 protein levels (Figure 2E,F). These results clearly show that BPA-G induced expression of some key adipogenic markers at both the mRNA and protein levels during adipocyte differentiation in 3T3L1 murine and primary human preadipocytes.

**Figure 3:**
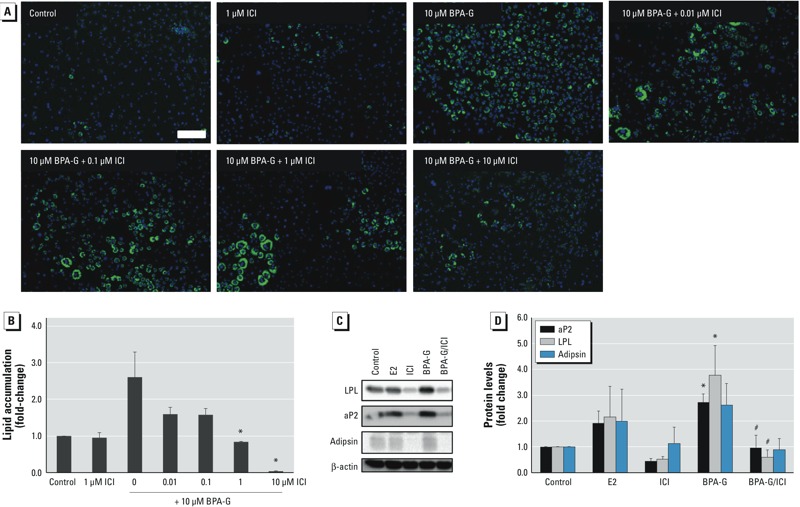
Effect of the ER antagonist ICI on BPA‑G–induced differentiation in 3T3L1 preadipocytes treated with ethanol (control), 1 nM estradiol (E2), or 10 μM BPA‑G in the presence or absence of increasing concentrations of ICI. (*A,B*) Lipid accumulation was visualized (*A; *bar = 200 μm) and quantified (*B*) by Nile red fluorescence. Images were taken on day 8 of differentiation. Images are representative of at least three separate experiments. (*C,D*) Protein levels of the adipogenic markers LPL, aP2, and adipsin in cells at day 8 of differentiation following treatment with E2, 10 μM BPA‑G, 1 μM ICI, or BPA‑G plus 1 μM ICI were assessed by Western blot (*C*) and densitometry (analysis (*D*). β-actin was used as the protein loading control. Values are expressed as mean ± SEM of six separate experiments. *p < 0.05 relative to control by one-way ANOVA with Holm-Sidak post-test analysis. ^#^*p* < 0.05 relative to BPA‑G alone.

**Figure 4:**
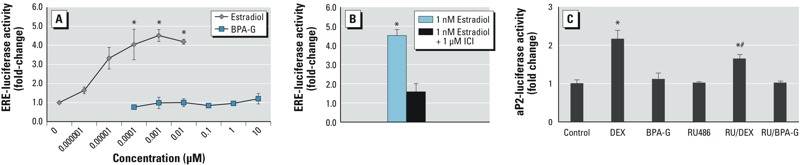
Effect of BPA‑G on ER and GR activity. (*A*) 3T3L1 preadipocytes were transfected with an ERE-luciferase plasmid and ERα and ERβ expression plasmids for 24 hr, then treated with increasing concentrations of estradiol or BPA‑G for an additional 24 hr; luciferase activity was then assessed. Values are expressed as mean ± SEM fold change of three separate experiments. *p < 0.005 relative to untreated control calculated by one-way ANOVA with Holm-Sidak post-test analysis. (*B*) 3T3L1 preadipocytes were transfected with an ERE-luciferase plasmid and ERα and ERβ expression plasmids for 24 hr then treated with 1 nM estradiol or 1 nM estradiol + 1 μM ICI for an additional 24 hr; luciferase activity was then assessed. Values are expressed as mean ± SEM fold change of three separate experiments. *p < 0.05 relative to untreated control by one-way ANOVA with Holm-Sidak post-test analysis. (*C*) Cos-7 cells were transfected for 24 hr with a GR expression plasmid (pTL2-GR) and either a 3X GRE-luciferase plasmid or with a reporter plasmid containing the promoter region of aP2 (aP2-luciferase). Cells were then treated with ethanol vehicle (control) DEX, or 10 μM BPA‑G with or without the GR antagonist RU486. Graphs illustrate fold change over control-treated cells. Values are expressed as means ± SEM fold change of three separate experiments. *p < 0.05 relative to control, and #*p* < 0.05 relative to DEX, by Student’s *t*-test.

*The effects of the ER antagonist ICI on BPA-G–induced differentiation*. Given that the parent compound (BPA) has ER binding activity at concentrations as low as 0.1 μM (Li et al. 2012), the potential role of the ER in BPA-G–induced differentiation was investigated using the specific ER antagonist ICI. We determined lipid accumulation in 3T3L1 cells treated with vehicle (control), 1 μM ICI, 10 μM BPA-G, or 10 μM BPA-G plus increasing concentrations of ICI. The data show that ICI alone did not induce significant lipid accumulation, whereas 10 μM BPA-G induced an increase to about 2.5-fold the level of control (Figure 3A,B). ICI significantly inhibited BPA-G–induced lipid accumulation at 1 and 10 μM ICI. Protein levels of adipogenic markers were also examined following treatment with 1 nM E2 or BPA-G with or without ICI. ICI significantly inhibited BPA-G–induced LPL and aP2 protein levels by 75% (to background levels) (Figure 3C,D). ICI also appeared to inhibit adipsin expression; however, the decrease in protein levels was not statistically significant due to greater variation in the Western blots. ICI or E2 alone had no effect on differentiation, consistent with previous reports (Boucher et al. 2014).We next confirmed that BPA-G does not have estrogenic activity using cells transiently transfected with an ERE-luciferase reporter plasmid. Data show that E2, used as a positive control, significantly stimulated ERE-luciferase activity at concentrations of 0.0001–0.01 μM, whereas BPA-G had no effect on ERE-luciferase activity (Figure 4A). The ability of ICI to inhibit E2-dependent luciferase activity was confirmed (Figure 4B).

*The effects of BPA treatment on GR transcriptional activity*. Cos-7 cells transfected with GR and a GRE-luciferase reporter plasmid were treated with either BPA-G or DEX, and luciferase activity was quantified. The data show that the GR agonist DEX was able to significantly induce luciferase expression; however, treatment of cells with 10 μM BPA-G had no effect on GRE-controlled luciferase activity (Figure 4C). An aP2-promoter reporter assay was also used to evaluate the effect of BPA-G on the ability of GR to regulate the aP2 promoter. DEX significantly stimulated aP2 luciferase activity in the presence of GR, and this effect was inhibited by co-treatment with the GR-antagonist RU486 (Figure 4C). However, treatment of cells with BPA-G in the presence of GR did not increase aP2 luciferase activity. Neither RU486 alone or BPA-G with RU486 co-treatment had any effect on GR-mediated aP2 luciferase activity. The effect of RU486 on BPA-G–mediated adipogenic marker expression was also evaluated by Western blots (see Supplemental Material, Figure S2). RU486 alone up-regulated protein levels of LPL, aP2, and adipsin, as does BPA-G; however, co-treatment of BPA-G and RU486 had no inhibitory effect. Therefore, it is unlikely that BPA-G–induced adipogenesis is mediated via the GR.

## Discussion

To our knowledge, the present study is the first to show that BPA-G is not an inactive metabolite, as previously believed, but is in fact biologically active and can induce lipid accumulation and differentiation of preadipocytes in murine and primary human cell models. It has been the long-held belief that the predominant metabolite of BPA, BPA-G, is inactive and quickly eliminated *in vivo*. However, the only studies that specifically examined the effects of BPA-G on any cellular or physiological response showed that it had no estrogenic activity, unlike its free precursor BPA, which is well known to have weak estrogenic properties (Matthews et al. 2001). However, BPA itself has been shown to be much more than just an estrogenic compound able to influence several signaling pathways such as GR, ERRs, androgen receptor, and TR, in addition to the traditional ERs (Lee et al. 2003; Matsushima et al. 2007; Moriyama et al. 2002; Sargis et al. 2010). Also, we and others have previously shown that BPA can induce differentiation of preadipocytes in rodent and human models (Boucher et al. 2014; Sargis et al. 2010; Wang et al. 2013). Only one study that we are aware of suggested that BPA-G may have a physiological effect (Weinberger et al. 2014); however, that study reported the effect of total BPA (both free BPA and BPA-G) and did not distinguish between the two forms. Weinberger et al. (2014) showed that total BPA concentrations in pregnant mothers were associated with reductions in gestation likely due to BPA/BPA-G–induced alterations in signaling via PPARγ or androgen precursors during pregnancy, suggesting an effect on the key adipogenic factor PPARγ. Therefore, it is possible that BPA-G may also bind and exert effects through these other receptors. The fact that we found BPA-G to be an active compound in adipogenesis suggests that it may have effects on other cellular processes warranting further study because BPA-G is the predominant metabolite following BPA exposure.

In the present study, we observed that BPA-G induced adipocyte differentiation and mRNA expression of the key adipogenic factors *SREBF1* and *LPL,* as well as aP2, LPL, and adipsin protein levels. We also found that BPA-G increased protein levels of the adipocyte marker aP2 in primary human preadipocytes. Future work will further characterize the effect of BPA-G on adipogenesis in these human cells. Consistent with previous reports, we also observed no ER-mediated transcriptional activity of BPA-G. Interestingly, the increase in mRNA expression of the key adipocyte transcription factors *CEBP*α and *PPAR*γ in response to BPA-G was not statistically significant, unlike the increase in *SREBF1* and *LPL* expression, both of which play important roles in the determination of the mature adipocyte phenotype. The concentrations of BPA-G used in this study are within the range of those used in other *in vitro* studies and found in human and animal fluids (Kosarac et al. 2012). BPA-G has been measured in urine of newborn infants in a small study of 12 infants, with an average concentration of approximately 2 nM (0.87 ng/mL) (Nachman et al. 2013), and was also detected (along with free BPA and BPA sulfate) in midgestation fetuses in a U.S. population of pregnant women (Gerona et al. 2013). Harthé et al. (2012) also reported BPA-G in human urine samples, with an average concentration of 11.5 nM (4.64 μg/L), similar to the *in vitro* concentration of BPA-G reported in ER-responsive transcription assays (Matthews et al. 2001) and the concentration used in this study.

Free BPA has been shown to activate both ERα and ERβ (Hiroi et al. 1999) as well as ERRγ (Okada et al. 2008). Previous studies found that BPA-G did not bind human ERα or ERβ *in vitro* nor stimulate ERα or β activity in MCF-7 cells at concentrations of 10 μM (Matthews et al. 2001). Consistent with these reports, we observed that BPA-G did not stimulate ER-responsive activity in 3T3L1 preadipocytes. Surprisingly, BPA-G–induced adipogenesis was inhibited by the ER-antagonist ICI at 1 and 10 μM concentrations. At 10 μM ICI, lipid accumulation appears to be inhibited even below background levels (Figure 3A/B); however, at such a high concentration, ICI likely has non-specific effects that may affect the ability of the cells to differentiate. Furthermore, all experiments were completed in 10% FBS, which contains some estrogen and estrogen-like compounds. Therefore, ICI would be expected to result in a slight inhibition compared with control. We previously reported that ICI was able to inhibit BPA-induced adipogenesis in primary human preadipocytes (Boucher et al. 2014) despite the fact that E2 did not have a positive effect on adipocyte differentiation and is considered to be antiadipogenic (Okazaki et al. 2002). Interestingly, it has been shown that diethylstilbestrol, a potent ERα activator, was adipogenic in mice and 3T3L1 cells and that the effect could be inhibited by ICI (Hao et al. 2012). The ability of ICI to inhibit the effects of free BPA on ERα and ERβ activation was also reported in HepG2 and HeLa cells (Li et al. 2012). The data therefore suggest that BPA-G may assert effects on adipogenesis through an indirect interaction with the ER pathway or an as yet unidentified nuclear receptor that can be inhibited by ICI. One other possibility is that BPA-G may be regulating adipogenesis via nuclear receptors from the ERR family, which are known to have a role in adipogenesis (Ijichi et al. 2007). ERRα has been shown to up-regulate PPARγ coactivator-1α (PGC-1α) expression and PPARγ/PGC-1α and SREBF1/PGC-1α dimers, which are important adipogenic transcription factors (Ju et al. 2012). Moreover, free BPA has been shown to bind strongly to ERRγ (Abad et al. 2008; Matsushima et al. 2007). However, the ability of BPA-G and ICI to affect the activity of ERR proteins has yet to be determined. Some reports have also suggested that free BPA mediates adipogenesis through binding and activation of the GR (Sargis et al. 2010), and we have shown that BPA up-regulated adipogenesis in the absence of glucocorticoids (Boucher et al. 2014). The present study clearly shows that BPA-G can partially replace the effect of DEX, a potent GR agonist, to induce the differentiation of 3T3L1 cells because differentiation is induced in the absence of a GR agonist. Moreover, GR-mediated up-regulation of aP2 promoter luciferase activity was not increased by BPA-G or inhibited by the GR-antagonist RU486, further suggesting that the effect is not mediated via GR. Free BPA has also been shown to bind TR and the androgen receptors (Lee et al. 2003; Matsushima et al. 2007; Moriyama et al. 2002), which may play roles in adipogenesis and could be potential binding partners for BPA-G, warranting further study. Only one study has examined binding of BPA-G to other cellular proteins, and that report evaluated the species-specific differences in transport and metabolism of BPA and BPA-G by rat and human ATP-binding cassette (ABC) transporters, which can interact with both chemicals (Mazur et al. 2012). Due to the importance of certain ABC transporters in cholesterol and phospholipid uptake and excretion, perhaps the binding of the transporter to BPA-G or free BPA may play a role in lipid metabolism and adipogenesis; however, this remains to be evaluated.

## Conclusions

To our knowledge, the present study shows for the first time that BPA-G is biologically active and promotes adipocyte differentiation and lipid accumulation *in vitro*. Our results indicate that while BPA-G did not have estrogenic activity, BPA-G–induced adipogenesis was still inhibited by an ER antagonist, suggesting the possibility that it may be acting through a nonclassical ER action or as yet unidentified pathway. Future studies will examine the potential roles of other nuclear receptors that might bind BPA-G and potentially activate adipocyte differentiation.

## Supplemental Material

(192 KB) PDF
