# Supplementary material for: In Vitro Effects of Bisphenol A β-D-Glucuronide (BPA-G) on Adipogenesis in Human and Murine Preadipocytes
Source: Environ Health Perspect. 2015 May 27;123(12):1287–93. doi: 10.1289/ehp.1409143 (PMC4671229; doi:10.1289/ehp.1409143)
Supplement: (192 KB) PDF [file ehp.1409143.s001.acco.pdf]

Environ Health Perspect
doi: 10.1289/ehp.1409143

Note to Readers: *EHP* strives to ensure that all journal content is accessible to all readers. However, some figures and Supplemental Material published in *EHP* articles may not conform to 508 standards due to the complexity of the information being presented. If you need assistance accessing journal content, please contact ehp508@niehs.nih.gov. Our staff will work with you to assess and meet your accessibility needs within 3 working days.

Supplemental Material

In Vitro Effects of Bisphenol A β-D-Glucuronide (BPA-G) on Adipogenesis in Human and Murine Preadipocytes

Jonathan G. Boucher, Adèle Boudreau, Shaimaa Ahmed, and Ella Atlas

Table of Contents

Figure S1. Time and dose-response of mRNA expression of adipogenic markers during differentiation. Differentiation and treatment of 3T3L1preadipocytes with increasing concentrations of BPA-G was induced as described. Total RNA was isolated on day 6 post-treatment and used for quantitative real-time PCR analysis of the adipogenic markers normalized to β-actin gene expression. Values are expressed as mean fold-change relative to control +/- SEM for 4 experiments.

Figure S2. The effect of the GR antagonist RU486 on BPA-G induced differentiation. 3T3L1 preadipocytes were treated with ethanol (control) or 10 µM BPA-G in the presence and absence of 1 µM RU486 and protein levels of the adipogenic markers LPL, aP2 and adipsin were assessed by Western blot (A) and densitometry (B) analysis at day 8 of differentiation following. β-actin was used as the protein loading control. Values are expressed as means +/- SEM for 3 separate experiments.

Figure S1

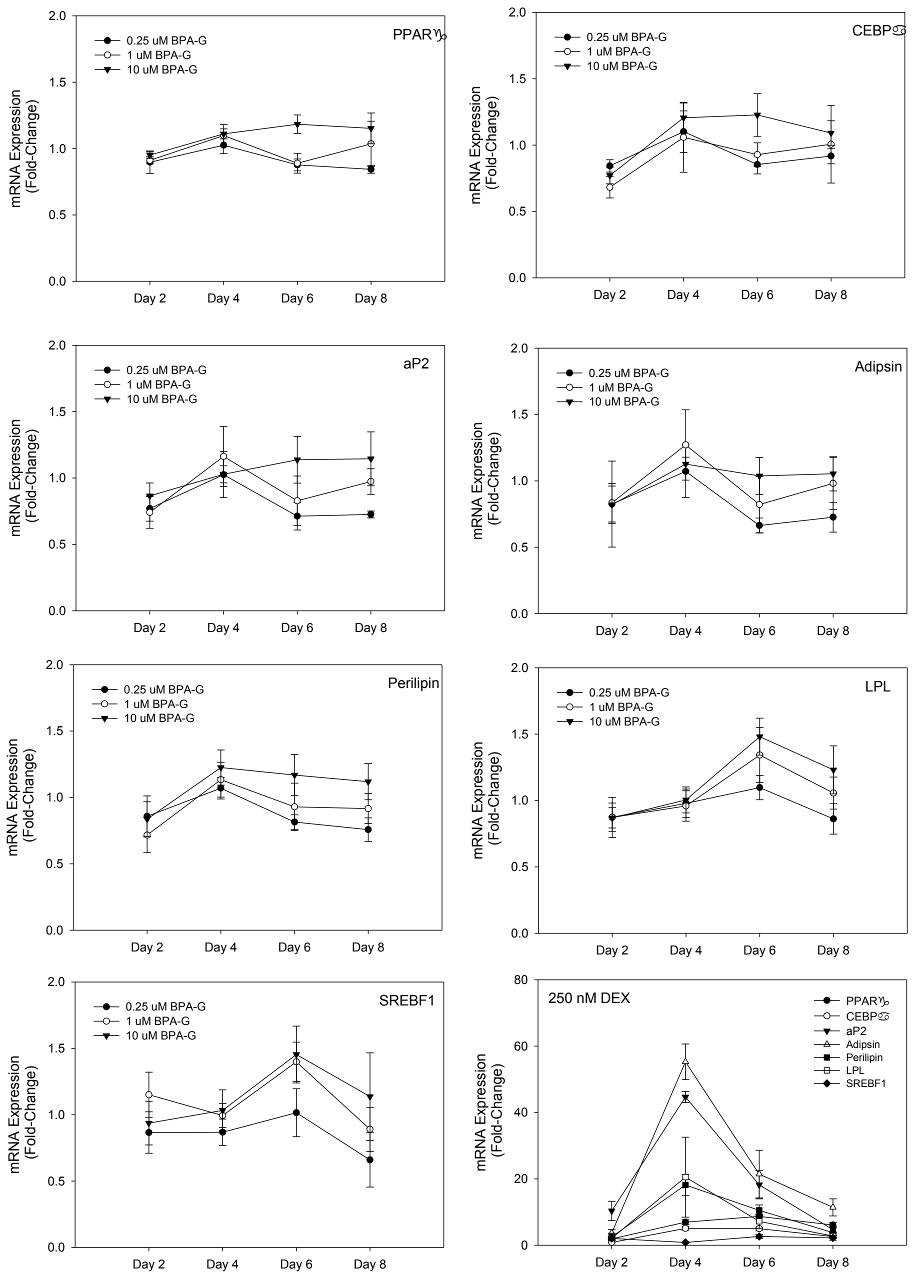

Figure S1. Time and dose-response of mRNA expression of adipogenic markers during differentiation. Differentiation and treatment of 3T3L1preadipocytes with increasing concentrations of BPA-G was induced as described. Total RNA was isolated on day 6 post-treatment and used for quantitative real-time PCR analysis of the adipogenic markers normalized to β-actin gene expression. Values are expressed as mean fold-change relative to control +/- SEM for 4 experiments.

Figure S2

A.

B.

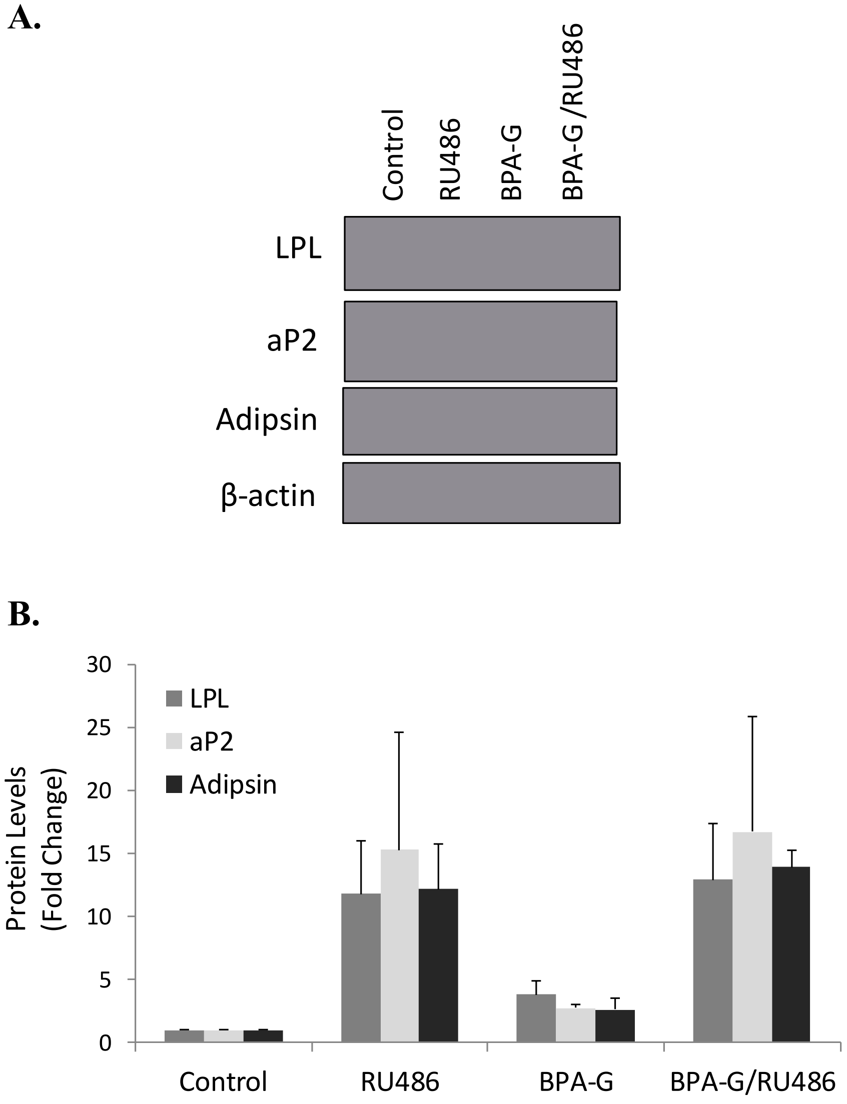

Figure S2. The effect of the GR antagonist RU486 on BPA-G induced differentiation. 3T3L1 preadipocytes were treated with ethanol (control) or 10 μM BPA-G in the presence and absence of 1 μM RU486 and protein levels of the adipogenic markers LPL, aP2 and adipsin were assessed by Western blot (A) and densitometry (B) analysis at day 8 of differentiation following. β-actin was used as the protein loading control. Values are expressed as means +/- SEM for 3 separate experiments.
